# Constipation

**Published:** 1904-10

**Authors:** 


### CONSTIPATION.*

It is fully appreciated that this subject, of so much importance both to patient and physician, can not be more than very briefly and superficially recounted in the short time allotted to the reading of papers before this society. Therefore no attempt is made to minutely detail either cause, symptoms or treatment, but to treat each in the more gross clinical aspect, yet with sufficient detail to point out the chief cause and to give the essential principles necessary to a successful treatment, because I am convinced no drugs, nor drugs are specifics, and each and every case holds something in common with every other case, hence there must be a common treatment for this trouble. Nor do I consider constipation from a surgical standpoint, leaving that to the specialist on rectal diseases, whose experience both from training and observation qualify him only as competent to attend to the trouble at that particular end.

First, be it understood that constipation is defined as sluggish or imperfect passage of feces through the tract, the degree depending upon the amount and duration of the more or less retention of this fecal mass. Second, that this fecal matter is composed of the undigested residue of the food in an admixture with the normal secretions and debris of the tract, the latter being no small amount, as is evidenced in disease when no nourishment is given from which a residue could be expected.

Some of the causes of constipation are improper food, improper amount of food, and the improper chemical action on the food during its passage through the tract.

What would be a nourishing food to one person could not be tolerated by another, the taste or after-effects of eating same being such that it would be refused. Yet, in general, what is good and proper food for one person is acceptable and nutritious to all, and *vice versa*. Circumstances and individual preference for some kinds of food often cause people to eat to excess of such, with a corres-

*Read by W. T. McKinney, M.D., before the Louisville Medical and Surgical Society, August 15, 190[illegible].

ponding manifestation of this excess, and likewise a showing of the want of other kinds, the eating of which had been neglected. This is often and easily done, as only a certain bulk of food can be taken, and if the necessary variety is not observed there are exhibited, as above stated, the symptoms of excess and lack. The system can only derive nourishment from food that it contains. The improper amount of food can be considered from the standpoint of either too much or too little.

In the process of metabolism at least sufficient food must be taken to replace waste and repair; over this amount is more or less, depending on the kind and amount, a draft on the energy of the system, and by working the economy over time, and using up the secretions, brings about further waste and utter fatigue of the secretions, hence a resulting inability to digest food and consequent constipation.

Too little food to supply waste and repair of course brings about an impoverished state. There is a loss of muscle tone, nerve force and blood discrepancies. Hence, again, a resulting inability to digest food and consequent constipation.

The improper action upon the food during its passage through the tract depends largely upon the condition of the tract, which we have seen can be brought about by too little or an excess amount of food. Again, any pathological condition that causes a loss of nerve force, muscular tone or disturbance in the character of the blood and secretions, must necessarily, sooner or later, cause an insufficient and improper handling of the food by the alimentary tract.

Food being absolutely essential to the normal condition of the body, and some error in the food or its handling being the cause for constipation, it is readily assumed that the cause for constipation is a dietary one, unless pathological conditions exist and are the cause.

The symptoms of constipation are both objective and subjective, yet are such in individual cases that no set symptoms can be laid down, each set of symptoms while unmistakable, yet belonging to particular causes, and responding to only the proper treatment.

The treament of constipation will vary with thé individual practitioner, depending upon his knowledge of the physiology of the digestive tract. In this, possibly more than in any other trouble, is it necessary to have the co-operation of the patient. A full explanation of the chemistry of digestion to an intelligent patient almost assures you success.

The first and essential step in the treatment is to revise the diet list. Many patients will tell you that they pass unchanged articles of diet, yet persist in eating them under the mistaken notion that such food is wholesome. Variety in food should be encouraged, and at least enough vegetables should be eaten to form residue; patients should be taught that while meats are nutritious they leave a small amount of residue. As to quantity, more trouble will be experienced in getting patients to eat enough than to control them from eating too much. It is rather the quality and the preparation of the food that causes trouble.

The administration of liquids should have the closest attention. Nature provided gastric juices in the proper quantity and the proper strength, and while liquids properly taken help to maintain this normal equilibrium, yet if improperly taken are often largely accountable for the resulting digestive disturbances.

Liquids should be taken not closer than one-half hour before meals nor one hour after meals. By the observance of this rule the gastrice juices are not diluted during and just after meals, when they have their task of digestion to perform. Water, which is the best liquid to drink, is always craved one or two hours after a meal taken without liquids, and at that time it is most useful, as it assists in the movement of the properly digested mass into the intestines.

Food taken with little or no fluids allows, in fact necessarily compels, better mastication, and obtains the benefit of ptyalin digestion to the food, of which it is of course robbed if every mouthful is washed down with liquid. Again, if food is to be taken dry, coffee, tea, cocoa and chocolate will be dispensed with, and their injurious effect lost. Between meals much water should be drank, every patient should be required to drink at least one-half gallon daily.

The above procedure will maintain perfect digestion in an otherwise healthy stomach, and also its strict observance will aid to bring about a normal condition in the stomach that has been required to handle an excess of liquids at meal times.

In the treatment of constipation, when indigestion is the cause, it is too often the stomach is treated when the gut is at fault. If the food is sufficiently masticated and the juices not too much diluted, no further attention to the stomach is usually required, and, too, there will be little or no disorder of the intestinal function, but if food passes into the intestines improperly prepared their function is impaired, and sooner or later constipation must ensue. Therefore, when digestants are given, such should be selected as will operate in the intestines, or at least must be added to those known to be only effective in the stomach. We may have imperfect gut digestion and perfect stomachic digestion, the result of insufficient secretions. This is generally remedied by the injection of sufficient water between meals.

To detail the drugs used and beneficial in the treatment of constipation would be entirely out of place; sufficeth it to say those used and results obtained will depend upon the diagnosis of cause.

If a digestant alone is needed we have a variety of good ones, both for the stomach and intestines.

If motor tone and vascularity is needed, strychnine is possibly our best remedy.

If secretions are to be increased, plenty of water between meals and the mineral acids after meals will give results sought.

In the matter of laxatives those that stimulate secretions in the entire tract should be selected.

I think it wise practice to have patients take a weekly saline laxative. This overcomes a sluggish bowel and improves assimilation.

Hand massage, following the normal direction of the fecal mass, is very beneficial.

Punctuality as to time of stool is productive of much good, yet it should be practiced intelligently. Going to stool at a certain time is not all that is required. Patients should be taught to dwell in thought upon the act from five to ten minutes before the time. This alone has been the means of largely increasing the value of an appointed time for stool.

The postponement of stool to a more convenient time can not be too severely condemned. The majority of observers think that the time for stool should be one when thoughts of other affairs should be excluded.

The patient should be taught to relax the muscles concerned in the act. There should be no strain, which only tends to contraction of the muscles and hinders the passage.

It should ever be borne in mind that agents used in the treatment of constipation are for the purpose of assisting nature to perform a normal function, and never for the purpose of having these agents do the work themselves. More attention should be paid to cause and its correction than to assisting digestion to combat the cause.—*American Practitioner and News.*

